# Mother's occupation and sex ratio at birth

**DOI:** 10.1186/1471-2458-10-269

**Published:** 2010-05-23

**Authors:** Kathreen E Ruckstuhl, Grant P Colijn, Volodymyr Amiot, Erin Vinish

**Affiliations:** 1 University of Calgary, Department of Biological Sciences, 2500 University Drive NW, Calgary, Alberta, T2N 1N4, Canada

## Abstract

**Background:**

Many women are working outside of the home, occupying a multitude of jobs with varying degrees of responsibilities and levels of psychological stress. We investigated whether different job types in women are associated with child sex at birth, with the hypothesis that women in job types, which are categorized as "high psychological stress" jobs, would be more likely to give birth to a daughter than a son, as females are less vulnerable to unfavourable conditions during conception, pregnancy and after parturition, and are less costly to carry to term.

**Methods:**

We investigated the effects of mother's age, maternal and paternal job type (and associated psychological stress levels) and paternal income on sex ratio at birth. Our analyses were based on 16,384 incidences of birth from a six-year (2000 to 2005 inclusive) childbirth dataset from Addenbrooke's Hospital in Cambridge, UK. We obtained a restricted data set from Addenbrooke's hospital with: maternal age, maternal and paternal occupations, and whether or not the child was first-born.

**Results:**

Women in job types that were categorized as "high stress" were more likely to give birth to daughters, whereas women in job types that were categorized as "low stress" had equal sex ratios or a slight male bias in offspring. We also investigated whether maternal age, and her partner's income could be associated with reversed offspring sex ratio. We found no association between mother's age, her partner's job stress category or partner income on child sex. However, there was an important interaction between job stress category and partner income in some of the analyses. Partner income appears to attenuate the association between maternal job stress and sex ratios at moderate-income levels, and reverse it at high-income levels.

**Conclusions:**

To our knowledge this is the first report on the association between women's job type stress categories and offspring sex ratio in humans, and the potential mitigating effect of their partners' income.

## Background

Psychological stress is omnipresent in our everyday lives. Studies have established links between 1) job stress and coronary heart disease [1,2] slower recovery from injuries [3], depression, and anxiety [4], 2) between maternal psychological stress and suppressed cell-mediated immunity [4], 3) between cortisol levels and preterm deliveries [5,6], or 4) between maternal psychological stress and early foetal abortions[6]. Offspring sex-ratio biases have been observed in human populations under a variety of stress-inducing circumstances such as economic collapse [7], earthquakes [8], caloric deprivation [9,10] and war [11,12]. Despite mounting evidence of the potential effect of stress on sex ratios in humans, very little is known about the mechanisms and ultimate causes for a shift in sex ratio at birth, and some of these have been questioned [12,13]. Proximate explanations for a shift in the sex ratio include the following: 1) differential sperm motility due to psychological stress in men (e.g. Kobe earthquake destroyed men's houses and killed their family members) [14,15], 2) Y-bearing sperm being faster but less resilient to unfavourable conditions in the mother's reproductive tract than X-bearing sperm, who are slower but survive longer [14,16], 3) spontaneous abortions may be biased towards males [16] and might in general be more common than abortions of female foetuses [17,18], 4) maternal psychological stress could lead to foetal asphyxia, foetal death, or complications during parturition [19,20].

Ultimate explanations in sex ratio changes include the Trivers-Willard hypothesis [18], which suggests that if a female is in poor condition, or of low social status, it is beneficial to her to invest into the offspring sex that is less reproductively variable. The reproductive success of male offspring, in a society where access to breeding partners is limited through dominance hierarchies and male-male competition, tends to be more variable and resource sensitive. Some males are thus highly successful breeders while others are not. Although females can benefit by investing into the offspring with higher reproductive variance they will not be able to do so if they lack the resources. Therefore, vertebrate females subjected to physiological stress, or in worse body condition gain a selective advantage by producing female offspring, since male offspring are thought to be more costly to produce and raise and are less likely to attain a high social status and lifetime reproductive success if born to a stressed, subordinate, female [18,21,22]. Support for a bias in sex ratios in humans comes, for example, from a study on women in a food-stressed rural community in southern Ethiopia, which showed that, women whose most recent offspring was male, had significantly higher body, fat, and muscle mass than other women, which led them to conclude that stronger mothers bear more sons than those in worse condition [23]. Hence, physiologically stressed females would be better off producing female offspring under conditions of stress, as daughters are more likely to survive than sons [23], and the reproductive success of daughters is usually not dependent on social status. One mechanism that would let females bias the offspring sex in their favour is the ability to abort males when the mother is in poor condition [9]. Then the female would have another opportunity to conceive a daughter, or a son in better condition, provided her own condition improved in the interim. Another assumption of the Trivers-Willard hypothesis [18] is that there is minimal parental investment by the male, beyond contributing sperm. This assumption is less applicable to humans, because males often invest parental care in their young, which reduces the variance of their reproductive success [18]. Many studies have been carried out trying to determine exactly how applicable the Trivers-Willard hypothesis [18] is to humans [24,25], and what kind of factors have an influence on the human sex ratio at birth [7,9,13,26-30]. Many researchers have found strong support for the Trivers-Willard hypothesis [30-34].

Although studies on the effects of physiological stress on offspring gender have been numerous, it is more difficult to investigate the link between psychological stress and offspring gender, since measures of psychological stress are inherently qualitative, rather than quantitative. It is thus not clear whether the Trivers-Willard hypothesis [18] applies to psychologically stressed women. There is some evidence that psychological stress can lead to changes in sex ratios in humans: A study on offspring sex ratios in Norwegian military air pilots [28] has shown that these pilots are more likely to have daughters than sons. Another study, of a British population, showed that women who had a lower perceived life expectancy were more likely to give birth to a daughter than to a son [35]. Despite an increasing number of women opting to have a career or to hold a job, and have a family, which can be extremely stressful, research on job types, their potential psychological stress levels and whether they could affect human sex ratio at birth is lacking. If chronic high levels of psychological stress were to affect a woman's reproductive processes, such as for example conception, or increase the risk of spontaneous abortions of male foetuses [4,5,16], we predict that women in job types that are labelled as " high stress", would be more likely to give birth to daughters than to sons, while women in "low stress" job categories should have a probability of giving birth to a son or daughter that is close to the world average. The world-wide proportion of male versus female births is at around 51.7% males and 48.3% females, or 105-107 males per 100 females [27], and between 104 and105 males per 100 females in the UK, during the time of this study http://www.statistics.gov.uk/STATBASE.

Other factors than psychological stress can also affect sex ratios and need to be included in analyses of the link between job stress and sex ratios in humans. Maternal age, for example, has been found to affect sex of offspring [36,37]. Younger women who have had at least one child, are more likely to produce boys [37], whereas women with a lower perceived life expectancy, or older women, might be more likely to have daughters [35]. Younger women who are presumably in better condition than those who are older, and women of higher parity have more vascularised uteri, which leads to infants of higher birth weight [37]. Almond and Edlund (2007) studied the effect of maternal age on offspring sex, and also found that younger women are more likely than older women to give birth to sons.

Parental economic status and education are useful measures of parental condition or the potential to invest resources into their children: individuals who earn more are more capable of satisfying their basic needs than those who earn less, and as a result will be in better condition. These assumptions are supported by the results of studies that have analysed the effects of parental economic status and education on offspring gender [24]. Koziel & Ulijaszek (2001) found partial evidence of greater investment in female offspring at the lowest level of paternal education and greater investment in male offspring at higher levels of paternal education. Similarly, a study on church rank in Mormons in the US found that women married to high ranking men were more likely to have sons than women married to lower ranking men [33]. Almond & Edlund (2007), who used education as one indicator of condition, found that better educated women had more sons. Lastly, the partner's own job type and associated psychological stress could have an effect on the total psychological stress experienced by women and therefore have an additive effect on the likelihood of giving birth to a girl or a boy. In this study, we thus investigated women's job types and associated stress levels, their age, and primiparity status (yes or no), their partners' job type and associated stress levels, their partner's income and whether or not these factors affected the sex ratio at birth. We hypothesized that all of these factors would influence the sex ratio at birth. We predicted that women with job types in the high stress category would be more likely to give birth to a daughter than a son, and that maternal age affects the sex of her offspring, biasing it towards daughters with older age. We also predicted that her partner's job stress and his level of income would affect sex at birth, with the expectation of a bias towards daughters for families with higher paternal stress and/or lower paternal income.

## Methods

Our study was based on 16,384 incidences of birth from a six-year (2000 to 2005 inclusive) childbirth dataset from Addenbrooke's Hospital in Cambridge, UK. Women, who were admitted to the hospital for delivery, were asked to fill in a form, which contained questions about their age, job, partners job, whether or not they already had children, etc. This form was stored and entered into a central database and information on the birth (gender, weight, health etc.) of offspring was added to this file. We obtained a restricted data set from Addenbrooke's hospital with: maternal age (mean = 30.94 years, SD = 5.34 years, Range = 13-53 years old, N = 16,345 women of known age), maternal and paternal occupations, and whether or not the child was first-born, a singleton or twin (we only looked at singleton births to avoid bias due to in vitro fertilization and possible hereditary effects). Consent to use and publish the data was obtained from the human ethics board of the University of Calgary, and the Addenbrooke's hospital (and Trust) in Cambridge, UK, after the nature and possible consequences of the studies were explained. All data were derived from anonymous subjects, whose identities were fully protected and cannot be revealed. If data were missing, about income or job description, or if there was no partner listed we excluded the data from the analyses. The study was conducted in the spirit of the Helsinki Declaration of 1975, as revised in 2000 (5).

We were unable to get a direct measure of maternal stress or paternal stress hormone levels (physiological stress). Instead we categorized job types into different levels of psychological stress by consulting several earlier studies that have assessed how stressful people perceive certain types of jobs to be (House 1974, the website of the Centre for Occupational and Health Psychology School of Psychology, Cardiff University's report (265/2000) and [38]. Jobs were categorized by lumping them into general job categories according to Marchand (2007), who had categorized job types according to how stressful people perceived their jobs to be. Marchand (2007) used stress levels between 1 and 10, with 1 equalling the lowest and 10 the highest amount of psychological stress (see Table 1). As an example, if the job type was described as "clerk" in a store, the job would be rated under the type "Sales, services" and given a stress rating of 5. Stay-at-home mothers and women working for the armed forces were not listed in Marchand's (2007) study. We decided to list these two professions under the "Health care" job category. The "Health care" job category in Marchand's (2007) study had a stress rating of 8 out of 10. The two occupations were added to that category because of the nature of the occupations: both are associated with high levels of stress, since they involve caring for dependents (similar to health occupations), or dealing with conflict. Hence, if the job were "night nurse" or "housewife" for example, then these jobs would both be placed in the "Health care" job type, which has a stress rating of 8. However, some people might argue that being a stay-at-home mother is a fairly stress-free job. We thus re-ran the analyses categorizing those mothers into stress level 2 instead of stress level 8, to see how it affected the sex ratio. Stress still had a highly significant, unchanged effect on the sex ratio, independent on where we put those mothers. The most likely explanation for no effect of these mothers on sex ratio is that there were not too many stay-at-home mothers in this study. They thus had little impact compared to the thousands of other mothers with a job type that was easily assigned to one of the stress levels. We thus decided to run the full model with all explanatory variables on two sets of data: the first with stay-at-home mothers having stress level 8 and the second where they had a stress level 2. In the end, we had 10 general job categories with associated stress levels, shown in Table 1.

**Table 1:** Job type stress levels and sex ratio.

Job category	Stress level	% male births	N
Arts, cultural, recreational, sports	1	52.10	119

Social, legal, government, educational, religious services	2	53.83	1257

Engineering, natural sciences, architectural, IT	3	49.02	714

Business, finance, administration	4	50.33	2492

Sales, services	5	51.47	3674

Management	6	51.61	2939

Farming, fishing and natural resources	7	44.90	49

Health, Stay-at-home, armed forces	8	52.34	4471

Trades, transport, construction	9	40.38	265

Processing, manufacturing, utilities	10	51.53	392

Given are different job type stress levels, percent male births, and sample sizes (N). Stress level of 1 = lowest stress level, 10 = highest stress level. The data set includes births and other information collected by Addenbrooke's hospital in Cambridge, England, between 2000 and 2005. P-values in bold indicate significant of p < 0.05 or smaller.

The partner's job type stress level was assigned using the same criteria as in Table 1. The partner's average income was calculated using information gathered by the 2007 Annual Survey of Hours and Earnings that was done by the UK government [39]. For our study, we used the median gross annual income for men in 2007, according to their occupation. In a second step, we investigated how maternal age, primiparity, the job type stress level, partner's job type stress level and income affected offspring sex at birth (see statistical analyses below).

In our statistical model, we included the following predictor (independent) variables: maternal job type stress level (ranging from 1 and 10), maternal age, partner job type stress level (ranging from 1 to 10) and income (in pounds sterling), whether or not the child was the first born, to investigate potential effects on sex ratio (dependent variable). We only included singleton births in the analyses. For this analysis we used an nominal logistic regression, which is the same as a generalized linear model, with a binomial distribution and a logit link function, using R statistical software (version 2.6.1; [40] and likelihood ratio tests [41] software. The nominal logistic regression allowed us to include several predictor variables that were either numerical (i.e. partner income) or categorical (stress levels), and produced odds ratios for the different levels of stress and sex ratio. Subsequently, we eliminated all non-significant variables from the model using a step-wise approach. Maternal age did not affect the likelihood of giving birth to a son or daughter (Logistic regression: Wald statistics = 0.42, p = 0.52, N = 16,335 births), but older mothers were more likely to occupy higher stress occupations (Logistic regression: Wald statistics = 84.07, p = 0.0001). In all subsequent models we left maternal age in as a covariate. We tested all other independent variables for collinearity but found none. In a final, more basic test, we compared the offspring sex ratio among women in job types with high stress indices (9 and 10) versus low stress jobs (stress levels 1 and 2), using a Likelihood ratio test (Chi-square), comparing numbers of occurrence.

## Results

### High versus low stress job types

Across the entire dataset, mothers in job types labelled as low stress (levels 1 and 2) were more likely to have a son than a daughter (53.68% sons), compared to mothers in job types labelled as high stress (levels 9 and 10) (47.05% sons, Chi-square = 7.85, p = 0.0051). Table 1 lists total number of births and sex ratio as a percent value.

### Association between job type stress levels and sex ratio, when stay-at-home mothers are considered to have a high stress occupation

**Figure 1:**
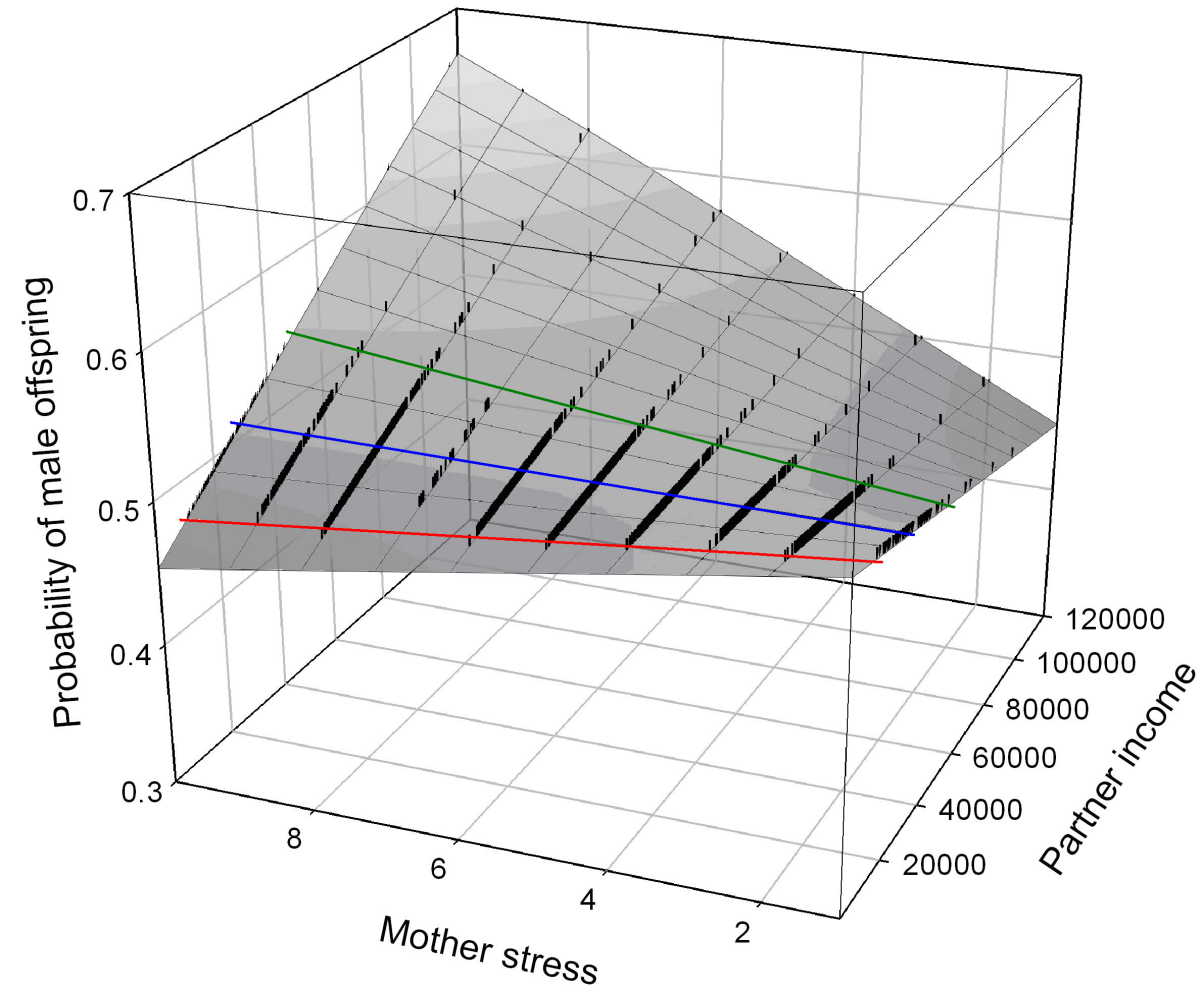
**Association between women's stress levels, partners' income and sex ratio at birth**. A response plane illustrating the effect of the interaction between mother's perceived job stress (x-axis) and partner income (z-axis) on the probability of producing male offspring (y-axis) for parents from Cambridge, UK from 2000 to 2005. Partner income is in £. The blue line illustrates the point (29,991 £) where the effect of partner income cancels the effect of mother stress, so that the probability of male offspring is equal across all levels of mother stress (probability = 0.509). The area between the red and blue lines is where higher levels of maternal stress decrease the probability of having a male. The area between the blue and green lines is where increasing partner income increases the probability of having a male. The individual black dots represent individual data points.

In the model with job type stress levels ranging from 1 (lowest) to 10 (highest), and with stay-at-home mothers in stress category of 8, neither partner income (Likelihood ratio χ^2 ^= 1.08, p = 0.2978), nor partner job stress (Likelihood ratio χ^2 ^= 14.7224, p = 0.0988) had a significant effect on the probability of having male offspring, but both maternal job type stress level ((Likelihood ratio χ^2 ^= 19.87, p = 0.0187) and the interaction of maternal job type stress and partner income ((Likelihood ratio χ^2 ^= 18.53, p = 0.0295) did have a significant effect (see Table 2 for odds ratios for different stress levels of mothers and the odds of having a boy or a girl). As maternal job type stress levels increased, the probability of having male offspring decreased. When plotting the interaction of maternal job type stress levels and partner income, it was evident that the majority of the partners fall into either the low or moderate income levels, with few at the higher income level (Fig. 1). When partner income was less than 29,991 £, increased maternal job type stress levels decreased the probability of male offspring (Fig. 1). At 29,991 £, the effect of partner income cancelled the effect of maternal job type stress, and there was an equal probability of male or female offspring at all maternal job type stress levels, which is 0.509 (Fig. 1). At more than 29,991 £, there was an inversion of the effect of maternal stress (Fig. 1).

**Table 2:** Comparisons of different levels of maternal stress for the odds (odds ratio and reciprocal) of having female versus male offspring at birth.

Level1	/Level2	Odds Ratio	95% CI of the odds ratio
2	1	0.94	1.07

3	1	1.12	0.89

3	2	1.20	0.83

4	1	1.05	0.95

4	2	1.12	0.89

4	3	0.94	1.07

5	1	0.98	1.02

5	2	1.05	0.95

5	3	0.87	1.14

5	4	0.93	1.07

6	1	0.99	1.01

6	2	1.06	0.94

6	3	0.88	1.13

6	4	0.94	1.06

6	5	1.01	0.99

7	1	1.44	0.69

7	2	1.54	0.65

7	3	1.28	0.78

7	4	1.37	0.73

7	5	1.47	0.68

7	6	1.45	0.69

8	1	0.97	1.03

8	2	1.04	0.97

8	3	0.86	1.16

8	4	0.92	1.08

8	5	0.99	1.01

8	6	0.98	1.02

8	7	0.67	1.49

9	1	1.54	0.65

9	2	1.64	0.61

9	3	1.37	0.73

9	4	1.47	0.68

9	5	1.57	0.64

9	6	1.55	0.64

9	7	1.07	0.94

9	8	1.59	0.63

10	1	0.82	1.22

10	2	0.87	1.14

10	3	0.73	1.37

10	4	0.78	1.28

10	5	0.83	1.20

10	6	0.83	1.21

10	7	0.57	1.76

10	8	0.84	1.18

10	9	0.53	1.88

The data set includes information on all births collected between 2000 and 2005 by Addenbrooke's hospital in Cambridge, UK. Level 1 indicates the stress level level2 is compared to. Stress levels range from 1 (low) to 10 (highest). Stay-at-home mothers were ranked in job stress level 8. CI = confidence interval.

### Association between job type stress levels and sex ratio, when stay-at-home mothers are considered to have a low stress occupation

When stay-at-home mothers were put in stress category 2, only the women's job type stress level significantly affected sex ratios in favour of girls (Likelihood ratio χ^2 ^= 28.8990971, p = 0.0007), while no other factor was significant (Table 3, for odds ratios).

**Table 3:** Comparisons of different levels of maternal stress for the odds (odds ratio and reciprocal) of having female versus male offspring at birth.

Level1	/Level2	Odds Ratio	95% CI of the odds ratio
2	1	0.90	1.11

3	1	1.11	0.90

3	2	1.23	0.81

4	1	1.06	0.94

4	2	1.17	0.85

4	3	0.95	1.05

5	1	1.01	0.99

5	2	1.12	0.90

5	3	0.90	1.11

5	4	0.95	1.05

6	1	1.00	1.00

6	2	1.11	0.90

6	3	0.90	1.11

6	4	0.95	1.06

6	5	1.00	1.00

7	1	1.31	0.76

7	2	1.45	0.69

7	3	1.17	0.85

7	4	1.24	0.81

7	5	1.30	0.77

7	6	1.30	0.77

8	1	0.91	1.10

8	2	1.00	1.00

8	3	0.81	1.23

8	4	0.86	1.17

8	5	0.90	1.11

8	6	0.90	1.11

8	7	0.69	1.44

9	1	1.57	0.64

9	2	1.74	0.58

9	3	1.41	0.71

9	4	1.48	0.67

9	5	1.56	0.64

9	6	1.57	0.64

9	7	1.20	0.83

9	8	1.73	0.58

10	1	1.00	1.00

10	2	1.11	0.90

10	3	0.90	1.11

10	4	0.95	1.06

10	5	0.99	1.01

10	6	1.00	1.00

10	7	0.77	1.31

10	8	1.11	0.90

10	9	0.64	1.57

The data set includes information on all births collected between 2000 and 2005 by Addenbrooke's hospital in Cambridge, UK. Level 1 indicates the stress level level2 is compared to. Stress levels range from 1 (low) to 10 (highest). In this comparison stay-at-home mothers were in stress category 2 (low stress). CI = confidence interval.

## Discussion

Our study showed a clear association between mothers' job type stress levels and the sex ratio at birth. As predicted, job types labelled as high stress were associated with a bias in sex ratios towards daughters in both analyses (stay-at-home mothers at low or high stress levels). Partner income itself was not a significant variable in explaining sex ratios but in the analyses with stay-at-home mothers in the higher stress category, 8, we found an interaction of partner income and maternal job stress on sex ratio. When partner income was high (specifically above 29,991 £), the sex ratio of offspring was biased towards sons, as predicted. However, when partner income was less than 29,991 £, the level of maternal job type stress had the biggest effect on the probability of having male offspring. Thus it seems, that the economic and social status, of males, are important in alleviating effects of women's occupational stress levels on offspring sex ratio.

Similarly, social status (wealth, church rank, and number of other wives) was reported to be an important factor in contributing to sex ratios in a Mormon community in the US [30]. Women who's husbands were high ranking in the church, rich, or who's husbands had several wives, were more likely to bear sons than women married to lower status men. Why this effect was only evident when stay-at-home mothers were put in a high stress category is unclear. In any case, partner income did not have any effect in our alternate model with stay-at-home mothers in stress category 2.

Analyses and assessment of stress levels for job categories and particularly subcategories in our study were to a large extent arbitrary as we were not able to measure stress levels directly but had to resort to studies about perceived stress levels in different job types. It would be very interesting to measure and monitor physiological stress levels directly, in different women, working different jobs, rather than having to resort to potential psychological job stress levels. Experiments done on starlings (*Sturnus vulgaris*), for example, indicate that physiological stress can bias the sex ratio at hatching [25]. Starling females, who were injected with stress hormones, had a higher proportion of female chicks than control females. Starling mothers had equal sex ratios in eggs laid but most of the male embryos died before hatching and the ones that hatched had low immune response and low survival, therefore biasing hatching sex ratio towards females. While such studies show that physiological stress can effectively bias the survival of one offspring sex over the other, we do not know whether psychological stress would have similar effects. Our results suggest that psychological stress could potentially have similar effects as we found that job types that were labelled as high stress were associated with a bias in the sex ratio in favour of daughters. The mechanisms behind this phenomenon, however, remain unclear. Evidence for both pre- and post-coital mechanisms exists but further research in this area is needed to investigate the potential pathways in more detail.

## Conclusions

Understanding what can bias the human sex ratio is especially important because there are already a number of existing factors, which influence sex ratio, and a number of new factors, which are just beginning to be identified, such as latitude[29], floods or smog [42], global warming [43], extreme life events[26], and as shown in our own study, women's job stress. Stress during conception and pregnancy are therefore possible candidates responsible for the decreasing sex ratios observed in many Western countries [44], in which women opt to have families, a job, and a career.

## Competing interests

The authors declare that they have no competing interests.

## Authors' contributions

KER conceived of and led the study, acquired the permits and data set, worked on the data set, did most of the statistical analyses and writing of the paper. GPC, VA and EV worked on the data set, assigned stress categories, and income levels, did part of the analyses and contributed to some extent to the writing of this paper. All authors read and approved the final manuscript.

## Pre-publication history

The pre-publication history for this paper can be accessed here:

http://www.biomedcentral.com/1471-2458/10/269/prepub
